# A Unique Role of the Oncoplastic Breast Surgeon’s Approach to an Inframammary Proliferating Trichilemmal Cyst: A Case Report

**DOI:** 10.7759/cureus.74227

**Published:** 2024-11-22

**Authors:** Shubashri Jeyaratnam, Han Boon Oh

**Affiliations:** 1 Department of Hand Surgery, National University Hospital, Singapore, SGP; 2 Department of Surgery, Ng Teng Fong General Hospital, Singapore, SGP

**Keywords:** benign mass, breast cyst, oncoplastic breast surgery, rare breast mass, trichilemmal cyst

## Abstract

Trichilemmal cysts, also known as pilar cysts, are commonly found on hair-bearing surfaces, such as the scalp or hairline. These are cysts that form from hair follicles and are benign. We describe an unusual case of a middle-aged lady presenting with a longstanding left-sided inframammary mass that had started growing more in the last year prior to the presentation. She was referred to the breast surgery clinic for further evaluation. A magnetic resonance imaging (MRI) scan was done for further delineation, which was not distinguished, and histopathological correlation was suggested. A decision was made for en bloc surgical resection with oncoplastic principles, ultimately resulting in a good cosmetic outcome for the patient.

## Introduction

Cystic-looking cutaneous lesions are a common presentation in the surgical clinic, as they are a source of concern for patients and practitioners alike. Potential malignancy or cosmetic concern is often the reason for presentation to a surgeon, who takes on the role of addressing both. Trichilemmal cysts are the second most common mucocutaneous cysts, next to epidermal cysts, and are thought to be benign in nature. They are usually found on the scalp or on a hair-bearing area. Their presence on the torso or in close relation to the breast has rarely been described. Our patient presented with an inframammary trichilemmal cyst, which we had not encountered on our breast service prior. While one may suspect a benign cyst clinically, histopathology may differ, and there are rare cases where malignancy has been reported [1]. Mimickers of trichilemmal cysts have included cystic basal cell carcinomas [2]. There have also been reports of malignant proliferating trichilemmal tumours (MPTTs), which appear morphologically similar to squamous cell carcinomas [4]. However, these are exceedingly rare and account for less than 0.1% of skin biopsies [5]. Given the possibility of malignancy and to evaluate if this was related to the breast or a separate lesion, our patient was presented at a multi-disciplinary tumour board for discussion of management prior to surgical planning. The decision was for marginal excision and formal histopathology prior to deciding if further treatment was required, as an incisional biopsy or fine needle aspiration cytology may not have yielded significant information. Oncoplastic principles were utilised in the en bloc resection of the specimen, utilising the inframammary fold (IMF) to create an inconspicuous scar. The patient was happy with her outcome, and further clinical surveillance will be performed downstream in view of the fact that this is a rare lesion and there are no guidelines available for surveillance at present.

## Case presentation

**Figure 1:**
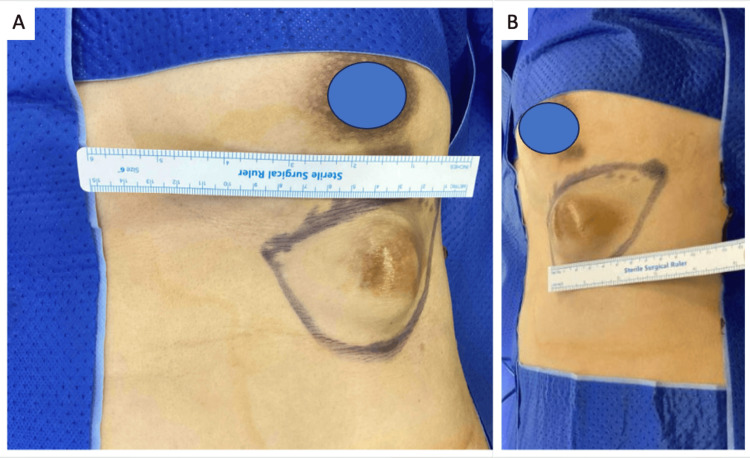
(A) Frontal view and (B) Lateral view of pre-operative markings and lesion

A 54-year-old Chinese female was referred to the specialist breast centre for evaluation of a 3 cm by 5 cm lesion situated at the IMF, at the junction of the breast and the left hypochondriac region. It was non-mobile, and no punctum was visible (Figure 1). The lesion had been present for over 10 years at the time of presentation and was concerning in view of interval growth in the last year, noticed by the patient. She had no family history of breast cancer or cystic lesions elsewhere and also had not highlighted the lesion to a medical practitioner prior. The lesion was painless, but she experienced some pressure symptoms from the bra line in view of the lesion’s location. There were no other lesions elsewhere, no nipple discharge, palpable breast lumps, or other significant findings of note. A pigmented naevus, inferior to the nipple, had been present since birth and was deemed distinct from the lesion of concern.

**Figure 2:**
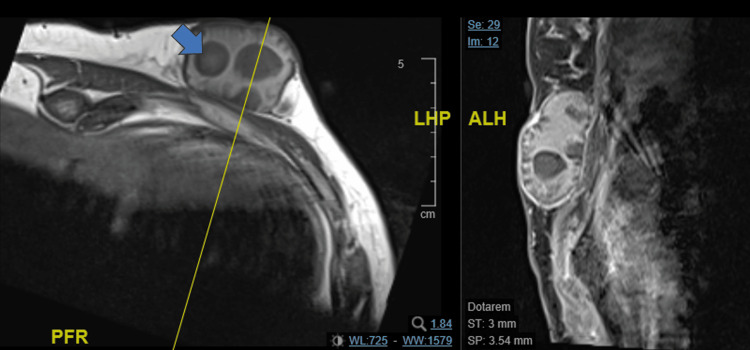
Axial and sagittal T1 weighted contrast MRI images of the lesion The solid component is depicted by an arrow with surrounding fluid MRI, magnetic resonance imaging

A mammogram (MMG) and targeted magnetic resonance imaging (MRI) were done for further delineation and to exclude a cutaneous adnexal cystic lesion of the breast. MMG was not significant, and targeted MRI revealed a well-circumscribed cystic lesion with T1w intermediate, high T2w hypointense solid papillary projections along the periphery. No contrast enhancement was seen in the internal solid components (Figure 2). The lesion was also noted to be superficial, from the muscle, and not extending into the thorax or involving the intercostals.

**Figure 3:**
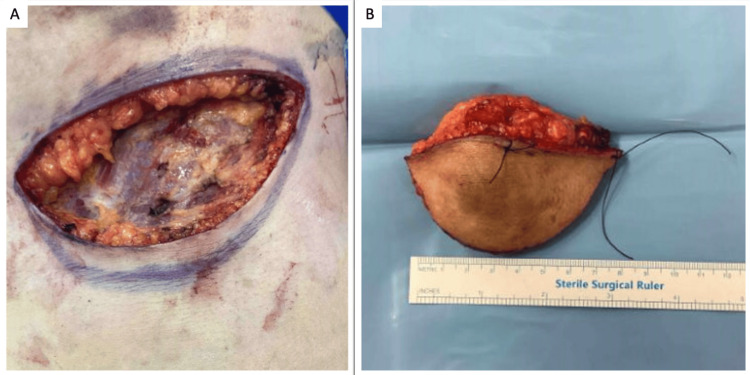
(A) Resection down to muscle with superior involvement of the inframammary fold and residual tissue defect and (B) Resected specimen

**Figure 4:**
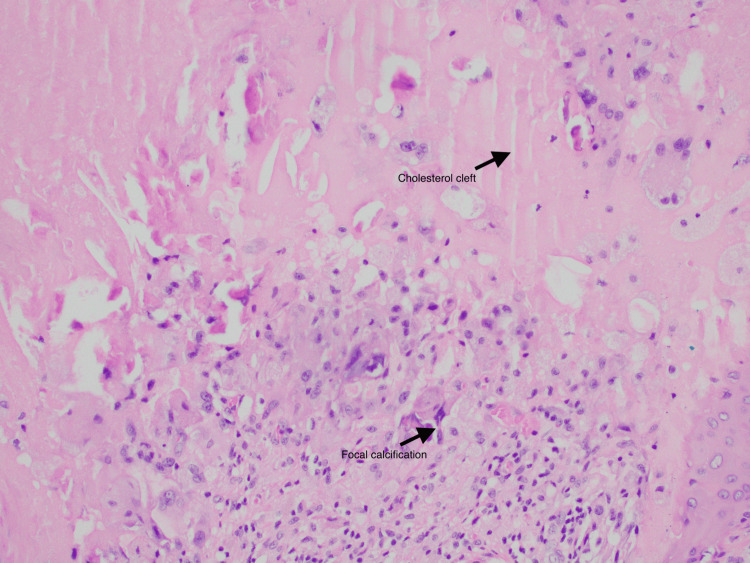
Representative histology slide with H&E staining at 20x magnification, demonstrating focal calcification and cholesterol clefts H&E, haematoxylin and eosin

Wide local excision (Figure 3) was performed via an inframammary incision, and histology reported by a dermatopathologist revealed clear margins of excision, with the cyst lined by proliferating epidermis with cystic infoldings devoid of any significant atypia or mitosis, and a granular cell layer absent at places. Cut sections revealed a 5.5 cm x 4.9 cm x 3.4 cm unilocular cyst directly beneath the hyperpigmented skin, containing greenish mucoid material and pasty tan-yellow material. No solid areas or papillary excrescences were seen. Microscopically, there was characteristic abrupt keratinisation, although no significant atypia or mitosis was visualised. Focal calcification and cholesterol cleft formation were also reported (Figure 4).

**Figure 5:**
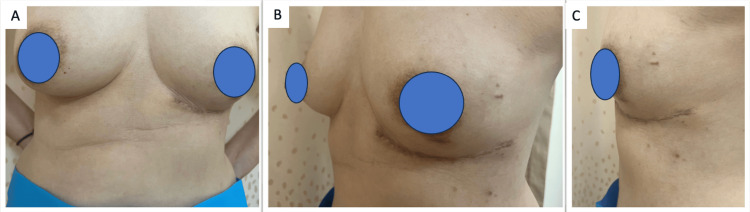
Final post-operative appearance, two weeks post-surgery (A) Frontal view, (B) Oblique view, and (C) Lateral view

The wound was closed with absorbable sutures and Dermabond® (Ethicon, Somerville, NJ, USA) glue, making use of and simultaneously preserving the IMF, with preferential undermining and advancement of the inferior abdominal wall skin. She was reviewed in the outpatient clinic, and the scar had healed flat and well, concealed within the bra line, with no significant IMF asymmetry (Figure 5). She is planned for a six-month follow-up for evidence of recurrence, where we will evaluate this clinically and potentially perform further imaging if any uncertainty exists.

## Discussion

Proliferating trichilemmal tumours (PTTs) are uncommon in a non-hair-bearing area, such as the breast or trunk. Most lesions are found on the scalp, with a particular predilection for women [6].

The presence of cytological atypia has been cited as a criterion for surveillance in view of their unpredictable prognosis, while those with no cytological atypia are known to behave in a benign manner [7]. Moreover, as core biopsies do not distinguish between benign and malignant proliferating trichilemmal cysts, and PTT to MPTT transformations, as well as the risk of recurrence and metastases, have been described [7-9], complete excision and histopathological correlation remain the mainstay. The existing literature on trichilemmal tumours over the breast is sparse. In our case, there was initial clinical ambiguity as to whether this lesion was extending from the breast or from the abdominal wall, as well as uncertainty regarding the depth of the lesion, which is why the relevant imaging was performed for pre-operative delineation. One case of a trichilemmal cyst of the breast in an elderly female, appearing as Breast Imaging Reporting and Data System 2 (BI-RADS 2) on an MMG, was described [10]. Another case of an MPTT with axillary involvement, subsequent to a total mastectomy and axillary dissection performed for an initial misdiagnosis of squamous cell carcinoma, was also reported [11].

There are no formal surveillance guidelines in place, given that trichilemmal tumours in or adjacent to the breast are rare. Until the presence of further literature, our recommendation would be for clinical evaluation similar to the triple assessment routinely done for breast lesions, and to present this at a multidisciplinary tumour board for discussion if clinical ambiguity exists. A triple assessment would comprise a history and physical examination, relevant imaging, and histology. Breast surgeons should be aware of this clinical entity and the potential for malignant transformation. A dermatopathologist should also review the histological specimen if this resource is available, as was done for our case. Surveillance visits and advice to the patient for self-surveillance should be rendered until more is known about tumour behaviour.

## Conclusions

Proliferating trichilemmal cysts of non-scalp origin are rare and possibly under-reported in the literature. Our case demonstrates the unique role of the oncoplastic-trained breast surgeon in facilitating an extensile incision that can be hidden in the IMF, with a small local advancement flap, as well as the multi-disciplinary input that guided decision-making. We recommend following up with the patient to look for clinical recurrence, and advising them on self-surveillance in the interim. This may be a good strategy to employ until further information on the subject matter exists.

## References

[1] Kamyab K, Kianfar N, Dasdar S, Salehpour Z, Nasimi M (2020). Cutaneous cysts: a clinicopathologic analysis of 2,438 cases. Int J Dermatol.

[2] Matsui K, Makino T, Watanabe H, Furuichi M, Hara H, Shimizu T (2009). Giant cystic basal cell carcinoma mimicking epidermal cyst. J Dermatol.

[3] Siddha M, Budrukkar A, Shet T, Deshpande M, Basu A, Patil N, Bhalavat R (2007). Malignant pilar tumor of the scalp: a case report and review of literature. J Cancer Res Ther.

[4] Alici O, Keles MK, Kurt A (2015). A rare cutaneous adnexal tumor: malignant proliferating trichilemmal tumor. Case Rep Med.

[5] Agarwal C, Pujani M, Raychaudhuri S, Arora S, Rana D, Chauhan V (2019). Squamous cell carcinoma versus malignant proliferating trichilemmal tumor: a histopathological dilemma with review of literature. Indian J Dermatol.

[6] Al Aboud DM, Yarrarapu SNS, Patel BC (2024). Pilar cyst. StatPearls [Internet].

[7] Adegun OK, Morley S, Kalavrezos N, Jay A (2019). Proliferating trichilemmal tumour: diagnostic challenge on core biopsy. BMJ Case Rep.

[8] Ye J, Nappi O, Swanson PE, Patterson JW, Wick MR (2004). Proliferating pilar tumors: a clinicopathologic study of 76 cases with a proposal for definition of benign and malignant variants. Am J Clin Pathol.

[9] Folpe AL, Reisenauer AK, Mentzel T, Rütten A, Solomon AR (2003). Proliferating trichilemmal tumors: clinicopathologic evaluation is a guide to biologic behavior. J Cutan Pathol.

[10] Pai N, Amonkar A, Ballal R (2017). An unusual presentation of a breast lump. Indian J Surg.

[11] Uchida N, Tsuzuki Y, Ando T (2000). Malignant proliferating trichilemmal tumor in the skin over the breast: a case report. Breast Cancer.

